# Quality Evaluation Research Based on Functional Component Group and Quality Control Coefficient: A Case Study of *Gardenia jasminoides*

**DOI:** 10.3390/foods15183346

**Published:** 2026-09-21

**Authors:** Pei Chen, Qinqin Zhang, Conglong Lian, Lu Xu, Jinxu Lan, Jun Liu, Suiqing Chen

**Affiliations:** 1 College of Pharmacy, Henan University of Chinese Medicihne, Zhengzhou 450046, China; chenp1113@163.com (P.C.); zhangqq2020123@163.com (Q.Z.); lian1988@hactcm.edu.cn (C.L.); 15939131537@163.com (L.X.); ianjx@163.com (J.L.); liujun_0325@163.com (J.L.); 2 Collaborative Innovation Center of Research and Development on the Whole Industry Chain of Yu-Yao, 156 Jinshui East Road, Zhengzhou 450046, China

**Keywords:** *Gardenia jasminoides*, functional component group, food ingredient, quality control coefficient, quality evaluation, chemical fingerprint

## Abstract

Quality is critical to the market competitiveness of medicinal and edible GF. However, the current quality standards of the Chinese Pharmacopoeia rely on a single index and cannot comprehensively evaluate food material with diverse origins and multiple functional components. This study established a holistic evaluation strategy based on “functional component group” and “quality control coefficient *Q*”. Seven stable fingerprint peaks of iridoids and iridoid glycosides were identified by chromatography, with *Q* ≥ 3.67 as the quality benchmark. In cell experiments, HQG outperformed QG in the MTT assay of LPS-stimulated RAW264.7 cells. In addition, 78 volatile compounds and 22 inorganic elements were analyzed by GC-MS and ICP-MS, respectively, as complementary analyses. Combined with chemometrics, the samples were discriminated according to geographical origin and tissue, and characteristic markers were screened on an exploratory basis. This study provides a scientific basis for precise quality control and screening of high-quality GF, and offers a reference framework for quality evaluation of other medicinal and edible homologous plants.

## 1. Introduction

*Gardenia jasminoides* Ellis. (GF), a plant belonging to the Rubiaceae family, is a typical dual-purpose resource with both a long history of medicinal use and significant modern value in the food industry. Globally, particularly driven by the rapidly growing consumer demand for natural and clean-label foods, GF has become a key raw material in the fields of functional food ingredients and natural colorants [1]. GF is rich in crocin-type compounds, a group of water-soluble carotenoids that are generally recognized as safe natural food colorants [2]. The major crocins of interest in gardenia are crocin-I, crocin-II, and crocin-III; crocin-IV and its aglycone crocetin are also involved. They are widely used for coloring beverages, bakery products, confectionery, spices, and processed meat products [3,4]. This not only meets the market demand for alternatives to synthetic colorants but also aligns with the global trend in the food industry toward transitioning to natural and healthy ingredients [5]. Studies have shown that the traditional Chinese medicine efficacy of GF corresponds to the anti-inflammatory, antioxidant, hepatoprotective, and choleretic activities of iridoid glycosides (e.g., geniposide and genipin-1-O-gentiobioside), as confirmed by modern research [6]. These components are associated with the core pharmacological effects of GF and, together with the chemical structures of iridoids and their glycosides, serve as key chemical markers for the “bitter and cold” medicinal property of the plant. These findings have elevated GF and its standardized extracts beyond the role of mere colorants, revealing significant potential in the field of functional food ingredients [7].

From a quality control perspective, the current Chinese Pharmacopoeia (2025 edition) only specifies that the geniposide content in dried gardenia fruit must not be less than 1.8% [8]. Although this standard plays an important role in ensuring basic safety and authenticity determination, it is a “minimum threshold” standard and thus cannot readily reflect the overall quality of the functional component groups, namely iridoid glycosides and crocins, nor can it distinguish raw materials that all meet the minimum standard but differ markedly in their internal chemical composition. Consequently, it cannot adequately support premium pricing for superior quality or refined product development. The chemical composition of gardenia fruit is also affected by geographical origin [9], cultivar, harvest maturity, and postharvest processing [10], and raw materials from different origins exhibit pronounced quality differences [11]. Therefore, it is necessary to establish a multidimensional, quantifiable quality evaluation strategy that takes into account both functional and commercial attributes.

In food chemistry and analytical science, comprehensively characterizing the chemical fingerprint of complex natural food ingredients while enabling authenticity verification and quality grading is a major methodological trend [12]. HPLC fingerprinting is powerful for assessing quality consistency because it provides an overall chemical profile [13]. However, traditional similarity analysis only gives vague “consistent/qualified” judgments and cannot support quantitative comparison or grading. Integrating fingerprint qualitative information with precise multi-component quantification and chemometrics has therefore become a mainstream approach [14], improving objectivity, discrimination, and prediction [15]. For medicinal and edible materials, quality and safety are multifaceted: besides major active or pigment components, volatiles influence flavor, consumer acceptance, and bioactivity [16], while inorganic elements may correlate with functional components. Thus, a multi-platform strategy combining major organic components, volatile aroma compounds, and macro-, trace-, and harmful elements can more comprehensively evaluate complex raw materials [17].

Fingerprint analysis with chemometrics can distinguish raw materials and origins [18]. Zhang et al. developed multi-wavelength fusion fingerprints for Gardeniae Radix [19], and Zhao et al. combined bar fingerprints with spectrum-effect relationships for rapid active-component screening [20]. However, these studies have not yet addressed the quantitative grading of fingerprint information, nor have they integrated multiple characteristic peak areas into a quantifiable grading coefficient. Using gardenia fruit as a case study, this study addresses the limitations of the current single-index standard by focusing on the efficacy-related iridoid glycoside functional component group, establishing a stable HPLC fingerprint, and, to the best of our knowledge, designing and validating for the first time a Quality Control Coefficient (*Q*). This model converts complex chromatographic information into an intuitive scalar, enabling quality grading of raw materials and providing new insights for raw material procurement and grading in the food industry. Subsequently, nine major active components were quantified in 43 batches of gardenia fruit samples from Henan, Jiangxi, Hunan, and Fujian; analysis of variance (ANOVA) and Orthogonal Partial Least Squares Discriminant Analysis (OPLS-DA) were combined to investigate origin-related differences and screen markers; Gas Chromatography-Mass Spectrometry (GC-MS) was used to preliminarily explore differential volatile markers for origin traceability; and Inductively Coupled Plasma Mass Spectrometry (ICP-MS) was used to analyse inorganic elements in different plant parts and to perform correlation analysis with functional components. These complementary analyses provide baseline data for processing, utilisation, and safety evaluation. This study not only addresses the quality control and traceability of gardenia fruit but also provides a transferable multidimensional quality evaluation and grading framework, offering a methodological reference for the quality systems of other complex natural food ingredients, spices, and medicine–food homologous substances.

## 2. Materials and Methods

### 2.1. Plant Material

For this study, the research group collected a total of 43 batches of GF samples from four provinces in China: Henan (T1–T18), Jiangxi (J1–J14), Hunan (H1–H6), and Fujian (F1–F5). The samples were collected independently from different batches. All samples were gathered during the same period to establish a quality control system for GF and to investigate differences in functional chemical components and quality characteristics among samples from different producing regions. Additionally, samples of *Gardenia jasminoides* var. radicans (GVR) were collected for comparative analysis. Between August and November 2023, GF samples from various harvest times were collected for analysis and validation. The harvest dates were designated as follows: TH1, 13 August 2023; TH2, 29 August 2023; TH3, 14 September 2023; TH4, 1 October 2023; TH5, 14 October 2023; TH6, 30 October 2023; and TH7, 15 November 2023. Three batches were collected at each time point, with approximately 15-day intervals between harvests. These samples were collected in Nanyang City, Henan Province, China. In November 2023, nine batches of samples from different colored peels and kernels of mature GF were collected from Tanghe County, Nanyang City, Henan Province. All samples were manually separated and labeled, with peels marked as P1-P4 and kernels as R1-R5. The samples were obtained from the same harvest batch. Detailed sample information is provided (Table S1). The samples were authenticated by Professor Suiqing Chen of Henan University of Chinese Medicine as *Gardenia jasminoides* Ellis., belonging to the Rubiaceae family.

### 2.2. Chemicals and Reagents

Reference standards of geniposide (No. 1) and crocin-I (No. 2) were purchased from Shanghai Yuanye Bio-Technology Co., Ltd. (Shanghai, China). Reference standards of genipin-1-O-gentiobioside (No. 3), gardenoside (No. 4), crocin-III (No. 5), and croceic acid (No. 6) were obtained from Beijing Zhongke Quality Inspection Biotechnology Co., Ltd. (Beijing, China). Reference standards of geniposidic acid (No. 7), crocin-II (No. 8), and crocin-IV (No. 9) were purchased from Sichuan Vicki Biological Technology Co., Ltd. (Sichuan, China). A C8–C20 n-alkane standard solution was purchased from Sigma-Aldrich (St. Louis, MO, USA). A multi-element standard solution containing Al, As, B, Ba, Ca, Cd, Co, Cr, Cu, Fe, Hg, Mn, Mo, Na, Ni, P, Pb, Sb, Se, Sn, Sr, Ti, Tl, V, and Zn, each at a mass concentration of 1000 μg/mL, as well as national standard samples of Ge, In, and Bi (1000 μg/mL), were purchased from the National Analysis Center for Iron and Steel (Beijing, China, Table S1). HPLC-grade acetonitrile and formic acid were supplied by Fisher Scientific (Fairlawn, NJ, USA). Ultrapure water (18.2 MΩ·cm) was prepared using a Milli-Q™ water purification system (Millipore, Milford, MA, USA). All other reagents were of analytical grade and purchased from China National Pharmaceutical Group Chemical Reagent Co., Ltd. (Shanghai, China).

### 2.3. Sample Preparation

#### 2.3.1. Preparation of Reference Standard Solutions for HPLC–SPD20A Detection

Standard reference solutions were prepared by completely dissolving compounds No. 1–9 from Section 2.2 in a 70% aqueous methanol solution, yielding reference standard solutions with the following concentrations (mg/mL): (1) 0.743, (2) 0.326, (3) 0.114, (4) 0.094, (5) 0.036, (6) 0.043, (7) 0.058, (8) 0.036, and (9) 0.041. Among these, compounds (1), (3), (4), and (7) were combined to form a mixed reference standard solution for iridoids and their glycosides, while compounds (2), (5), (6), (8), and (9) were combined to form a mixed reference standard solution for crocins. All solutions were stored at 4 °C prior to analysis.

#### 2.3.2. Preparation of Test Sample Solutions for HPLC–SPD20A Detection

All samples were pulverized and passed through a No. 4 sieve. A 0.3 g portion of the GF medicinal powder was placed into a 50 mL centrifuge tube. A 30 mL volume of 70% methanol was added, and the tube was weighed. The mixture was subjected to ultrasonication for 30 min, cooled to room temperature, and the lost weight replenished with 70% methanol. After thorough mixing, the mixture was centrifuged at 9500 r/min for 5 min. The supernatant was collected, filtered through a 0.22 μm microporous membrane, and the resulting solution was used as the test sample solution.

#### 2.3.3. Preparation of Reference Standard Solutions for GC-MS Detection

A C8–C20 n-alkane standard solution was used as the reference standard solution.

#### 2.3.4. Preparation of Test Sample Solutions for GC-MS Detection

Fifty grams of GF powder was weighed, soaked in 10 times its volume of water for 2 h, and then subjected to steam distillation for 5 h until no further increase in volatile oil was observed. During collection, 1 mL of n-hexane was added to dissolve the volatile oil. After dehydration and drying with anhydrous sodium sulfate, the solution was sealed and stored at 4 °C for later use.

#### 2.3.5. Preparation of Reference Standard Solutions for ICP-MS Detection

Standard reference solutions were prepared by taking 1 mL of the multi-element standard solution (1000 μg/mL) from Section 2.2, diluting it to 50 mL with 2% nitric acid, and mixing thoroughly to obtain a mixed standard stock solution with a concentration of 2000 μg/L. This stock solution was then further diluted with 2% nitric acid to prepare mixed standard solutions at concentrations of 1000, 500, 250, 50, and 10 μg/L. Using the same method, a mixed standard stock solution containing trace and heavy metal elements was prepared and diluted with 2% nitric acid to create multi-element mixed standard solutions at concentrations of 125, 100, 50, 25, 10, 5, and 1 μg/L. Precise volumes of Ge, In, and Bi single-element standard solutions were similarly prepared and diluted to 10 μg/L to serve as the internal standard solution.

#### 2.3.6. Preparation of Test Sample Solutions for ICP-MS Detection

Approximately 0.2 g of the GF sample was accurately weighed into a polytetrafluoroethylene (PTFE) digestion tube. A 6 mL volume of nitric acid was added, and the powder was thoroughly mixed with the liquid. The tube was placed in an electric acid evaporation apparatus and pre-digested at 130 °C for 15 min, then removed and cooled to room temperature. After tightening the lid, the tube was placed in a microwave digestion instrument. Digestion was performed according to the following program: ramp to 120 °C over 7 min and hold for 5 min; ramp to 150 °C over 5 min and hold for 10 min; ramp to 180 °C over 5 min and hold for 40 min. Upon completion of the program, the tube was uncapped and returned to the acid evaporation apparatus for acid removal at 110 °C for 40 min. It was then removed, diluted to 100 mL with ultrapure water, and filtered to obtain the final test solution. A background blank solution was prepared in the same manner without adding the sample powder.

### 2.4. Study of the Functional-Chemical Component Group

#### 2.4.1. Instrumentation and Measurement Conditions for HPLC–SPD 20A Detection

HPLC analysis was performed using a Shimadzu LC-20A chromatograph equipped with an SPD-20A ultraviolet detector(HPLC, Kyoto, Japan). Chromatographic separation was achieved using an Agilent Extend-C18 column (4.6 × 250 mm, 5 µm) maintained at 30 °C. For the analysis of iridoids and their glycosides, the mobile phase consisted of acetonitrile (A) and a 0.1% formic acid aqueous solution (B). The gradient elution program was as follows: 5% A (0–1 min), 5–9% A (1–5 min), 9–12% A (5–11 min), 12–19% A (11–20 min), 19–22% A (20–25 min), 22–27% A (25–32 min), 27–34% A (32–40 min), 34–5% A (40–45 min), and 5–5% A (45–50 min). The flow rate was set to 1.0 mL/min, the detection wavelength was 240 nm, and the injection volume was 10 µL.

#### 2.4.2. Methodological Validation

When evaluating the repeatability, stability, and precision of the analytical method using sample S2, the same sample was injected consecutively six times for precision analysis. For stability assessment, the same sample was injected into the HPLC system at 0, 2, 4, 8, 12, and 24 h. Six independently prepared test sample solutions from sample S2 were used to verify repeatability. Using geniposide as the reference peak, the relative retention time (RRT) and relative peak area (RPA) of the main common peaks were recorded. The conclusions of the methodological validation were based on the relative standard deviation (RSD) of the RRT and RPA.

#### 2.4.3. Establishment of the Chemical Fingerprint of the Functional Component Group

Based on the stipulation in the Chinese Pharmacopoeia (2025 edition) that the geniposide content in the iridoid and related component group must not be less than 1.8%, we introduced the concept of a “Functional Chemical Component Group.” The iridoids and their glycosides, which are related to the pharmacological effects of GF, were screened as the evaluation indicators to define the structure of this “Functional Chemical Component Group.” Subsequently, the data from all test sample solutions were imported into the “Similarity Evaluation System for Chromatographic Fingerprint of TCM (2012 version)” to establish the HPLC characteristic component fingerprint for GF and to identify the common peaks.

### 2.5. Quantitative Analysis

#### 2.5.1. HPLC Conditions

The HPLC conditions for the quantitative analysis of iridoids and their glycosides were identical to those described for the fingerprint analysis in Section 2.4.1. For the analysis of crocins, the mobile phase consisted of A (acetonitrile) and B (0.1% formic acid aqueous solution). The gradient elution program was as follows: 20–25% A (0–3 min), 25–29% A (3–5 min), 29% A (5–10 min), 29–33% A (10–13 min), 33–37% A (13–22 min), 37–45% A (22–32 min), 45–20% A (32–40 min). The flow rate was set to 1.0 mL/min, the detection wavelength was 440 nm, and the injection volume was 10 µL.

#### 2.5.2. HPLC Method Validation

Following the method described in Section 2.3, a series of mixed reference standard solutions at appropriate concentrations were prepared and measured. Precision and stability were assessed according to Section 2.4.2. Repeatability was tested by independently preparing six parallel sample solutions according to the established sample preparation procedure. Accuracy was evaluated using a recovery experiment via the standard addition method, in which a known amount of reference standard at the same concentration was spiked into the sample matrix. The spiked samples were then processed and analyzed following the same sample preparation and HPLC procedures.

### 2.6. GC-MS Analysis

#### 2.6.1. Chromatographic Conditions

An HP-5MS elastic quartz capillary column (0.25 mm × 30 m, 0.25 μm) was used with high-purity helium as the carrier gas. The injection volume was 1 µL. MS conditions: ionization mode, EI; electron impact energy, 70 eV; ion source temperature, 250 °C; solvent delay time, 2.5 min; scanning mode, full scan; and detection range, *m*/*z* 30–445 (GC-MS, Waltham, MA, USA).

#### 2.6.2. Calculation of Retention Index (RI)

The Retention Index (RI) was calculated using the formula: RI = 100Z + 100 × [tR(x) − tR(Z)]/[tR(Z + 1) − tR(Z)], where Z and Z + 1 represent the number of carbon atoms in the n-alkane homologues eluting immediately before and after the analyte, respectively. The Automatic Mass Spectral Deconvolution and Identification System (AMDIS) was used to process the Total Ion Chromatogram (TIC) of the samples. Compounds were accurately identified by comparing the calculated RI values with the RI reference values obtained by searching the NIST 14 mass spectral library. The chemical structure yielding the highest spectral similarity and closest RI value was selected as the best match.

#### 2.6.3. Component Analysis

Following the method described in Section 2.3, the volatile oil extracted from GF material and the C8–C20 n-alkane standard solution were analyzed under the conditions specified in Section 2.6.1 to obtain TIC data. The AMDIS software was used to process the TIC data, removing interference peaks to obtain relatively clean mass spectra. Compounds were accurately identified based on the calculated RI values. Preliminary work in the laboratory had identified 78 volatile components in GF. The relative quantification of volatile compounds from different geographical origins was performed using the peak area normalization method.

### 2.7. ICP-MS Analysis

#### 2.7.1. Instrumental Measurement Conditions

The instrument used was an iCAP Q (ICP-MS, Waltham, MA, USA). The analysis mode was KED (Kinetic Energy Discrimination). The sample introduction time was 60 s and rinse time was 30 s. Each sample was analyzed three times with three data acquisitions per peak.

#### 2.7.2. ICP-MS Method Validation

Validation was performed following the method described in Section 2.5.2, with ICP-MS used for measurement and analysis.

### 2.8. Cell Viability Assay

RAW 264.7 macrophages (Shanghai Cell Bank, Chinese Academy of Sciences) cultured to the fourth passage were seeded into 96-well plates at a density of 3 × 10^4^ cells per well. The cells were cultured overnight in high-glucose DMEM medium (Servicebio, Wuhan, China) supplemented with fetal bovine serum (FBS, Servicebio, Wuhan, China) in an incubator at 37 °C with 5% CO_2_. The next day, the old medium was discarded, and the cells were treated with 10 μg/mL of *Gardenia jasminoides* extracts of HQG (High-quality group, T3, *Q* > 3.67, geniposidic acid:genipin-1-O-gentiobioside:geniposide = 0.0792:0.2596:1) and QG (Qualified group, T10, *Q* < 3.67, geniposidic acid:genipin-1-O-gentiobioside:geniposide = 0.1618:0.5498:1), with six replicate wells per group. A control group (CON) was treated with medium only. A model group (M) was stimulated with 1 μg/mL lipopolysaccharide (LPS, Sigma-Aldrich, USA) for 24 h. After 24 h of culture, 20 μL of MTT solution (Beijing Solarbio Science & Technology Co., Ltd., Beijing, China) was added to each well, followed by further incubation for 4 h. The supernatant was carefully aspirated, and 150 μL of DMSO (Waltham, MA, USA) was added to each well. The plates were then shaken for 10 min to completely dissolve the formazan crystals. The absorbance (OD value) was measured at 490 nm using a microplate reader (Thermo Fisher Scientific, Varioskan™ LUX, VL0000D0, Waltham, MA, USA). Cell viability (%) was calculated as follows: (OD of treatment group − OD of blank group)/(OD of control group − OD of blank group) × 100%. Each independent experiment was performed in triplicate (Table S1).

### 2.9. Data Processing

Principal Component Analysis (PCA), Orthogonal Partial Least Squares Discriminant Analysis (OPLS-DA), and s-plot analysis were performed using SIMCA 14.1 software (14.1 version, Umetrics, Umea, Sweden). Statistical analysis and graphing were conducted using IBM SPSS Statistics (27 version), Origin Pro (2024 version), and GraphPad Prism (10 version). Statistical significance was determined using the t-test and one-way ANOVA. Similarity analysis of HPLC fingerprints was performed using the Similarity Evaluation System for Chromatographic Fingerprint of TCM (2012 version, China Pharmacopoeia Committee).

## 3. Results

### 3.1. Establishment of the Quality Control System

#### 3.1.1. Screening of Characteristic Peaks

**Figure 1:**
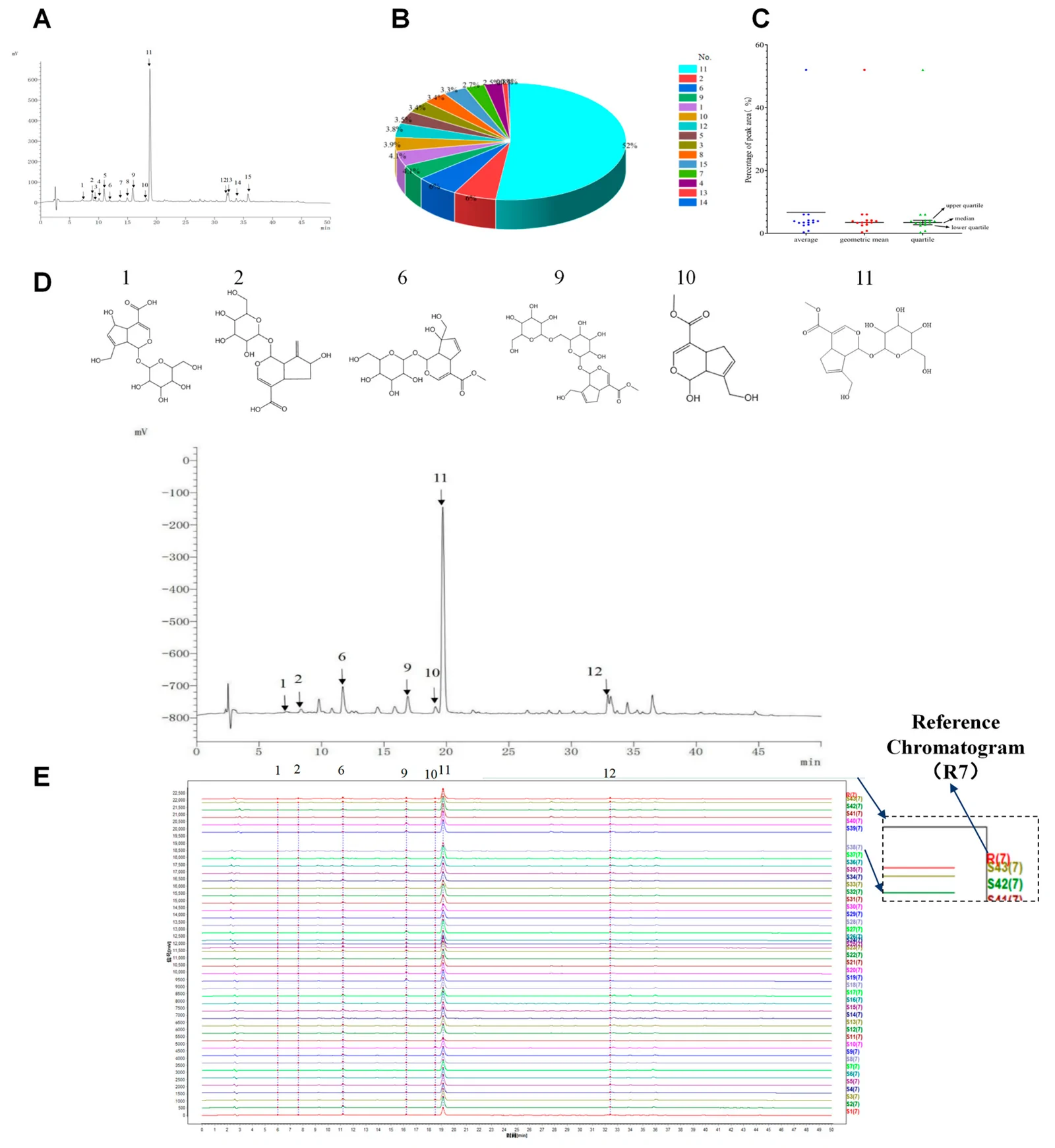
(**A**) Chromatographic profile of the iridoid and glycoside fraction of GF.The numbers represent the detected components. (**B**) Percentage contribution of the 15 chromatographic peaks in the iridoid and glycoside fraction of GF. (**C**) Distribution of peaks under three different selection methods. (**D**) Characteristic peaks of the iridoid and glycoside fraction of GF: 1, deacetylasperulosidic acid; 2, gardoside; 6, gardenoside; 9, genipin-1-O-gentiobioside; 10, genipin; 11, geniposide. (**E**) Characteristic peak fingerprint of the iridoid and glycoside fraction of GF. R7 is the reference chromatogram.

In preliminary work, the research group employed LC-MS (San Jose, CA, USA) technology to analyze and identify iridoids and their glycosides in GF. By comparing with reference standards and chemical identification results, the following peak information was ultimately obtained: Peak 1 corresponds to deacetylasperulosidic acid, Peak 2 to gardenidic acid, Peak 3 to shanzhiside, Peak 4 to geniposidic acid, Peak 6 to gardenoside, Peak 7 to feretoside, Peak 9 to genipin-1-O-gentiobioside, Peak 10 to genipin, and Peak 11 to geniposide (Figure 1A, Table S2). Among the nine identified peaks, the peak areas corresponding to iridoids and their glycosides accounted for 85% of the total peak area. Consequently, the chromatographic components obtained via this extraction method were confirmed to be the iridoids and their glycosides of GF.

Based on the data from the 15 absorption peaks, the area of each peak was ranked in descending order of its percentage contribution, and a chart showing the proportion of each peak was plotted (Table 1, Figure 1B). The results indicate that the proportion of peak area for iridoids and their glycosides ranged from 0.35% to 52.01%, showing significant variation. This study employed a comprehensive comparison using the geometric mean, positional mean, and upper and lower quartiles of the percentage contribution of each individual peak area to determine the optimal characteristic peaks (Figure 1C). The findings reveal that only one peak exceeded the positional mean, which was Peak 11, accounting for 52.01%. Additionally, seven peaks had percentage contributions exceeding the geometric mean position, namely Peaks 1, 2, 6, 9, 10, 11, and 12. Four peaks fell within the upper quartile range: Peaks 2, 6, 9, and 11; similarly, four peaks were within the lower quartile range: Peaks 4, 7, 13, and 14.

**Figure 2:**
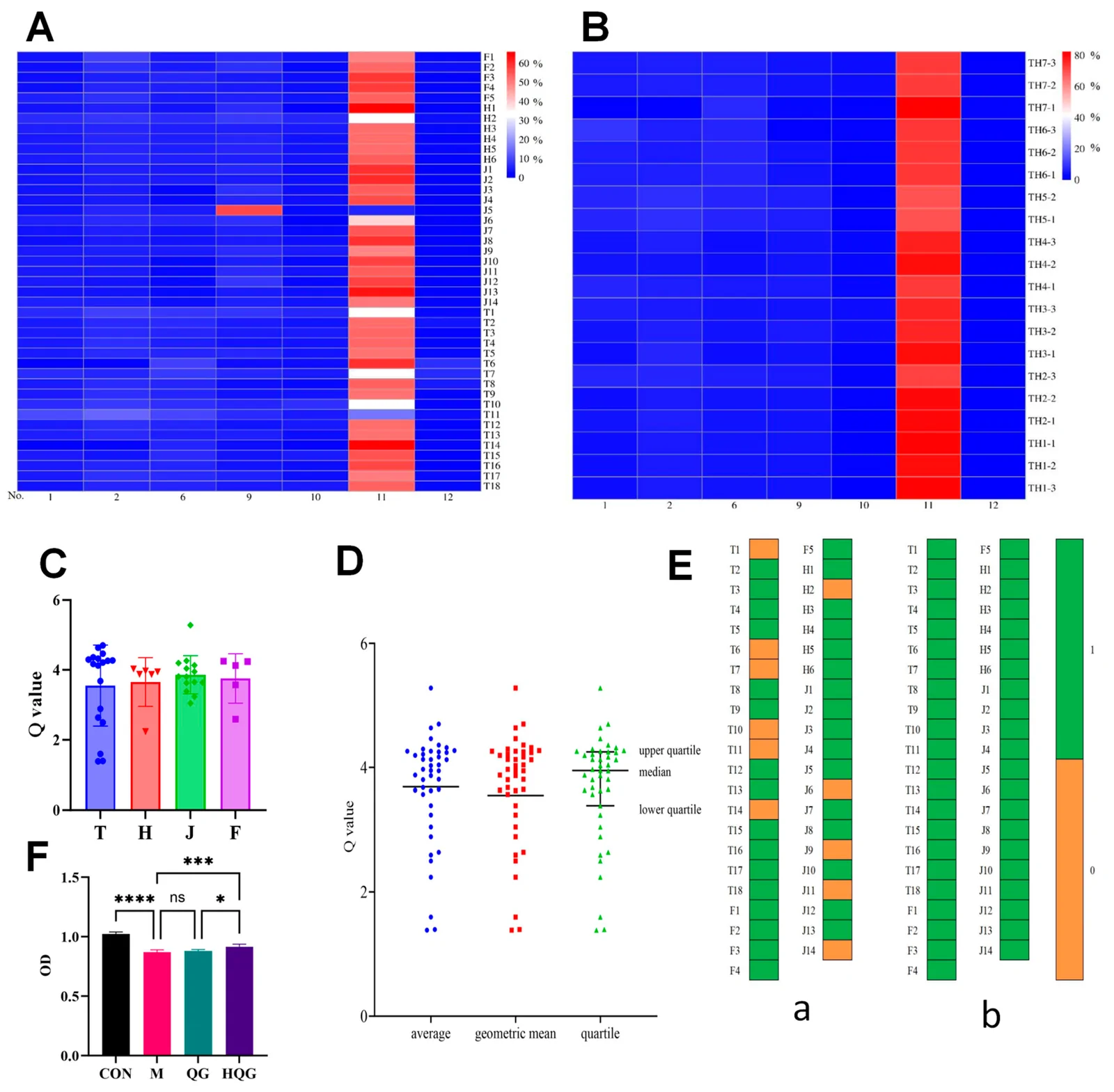
(**A**) Individual peak percentage contributions of characteristic peaks in the iridoid and glycoside fraction of GF from different geographical origins. (**B**) Individual peak percentage contributions of characteristic peaks in GF at different harvesting times. (**C**) Distribution of *Q* values for GF from different geographical origins (T: Henan origin; H: Hunan origin; J: Jiangxi origin; F: Fujian origin; the same applies below). (**D**) Screening results of *Q* values for the 43 batches of medicinal materials. (**E**) Comparison of results between the quality control coefficient standard and the content detection standard of the Chinese Pharmacopoeia (**a**: Quality control coefficient standard; **b**: Chinese Pharmacopoeia content detection standard). (**F**) Cell viability. * *p* < 0.05; *** *p* < 0.001; ns, no significant difference. Control group (CON), Model group (M), Qualified group (QG), High-quality group (HQG).

In summary, the upper quartile method resulted in a smaller number of selected peaks, so priority was given to methods yielding a greater number of peaks for reference. Based on the response signals of each chromatographic peak and the identified peaks, the geometric mean was selected as the reference line. Consequently, seven characteristic peaks for the iridoid glycosides and their related components in GF were ultimately screened. These characteristic peaks are Peak 1, Peak 2, Peak 6, Peak 9, Peak 10, Peak 11, and Peak 12. The results are shown in Figure 1D, with the sum of the areas of the selected characteristic peaks accounting for approximately 80.02% of the total peak area. On this basis, we constructed characteristic peak fingerprint profiles of iridoids and their glycosides from different geographical origins, calculated the percentage contribution of individual characteristic peaks, and performed visual characterization (Figure 1E and Figure 2A, Table S3). According to the existing HPLC-based quality control standards for GF, geniposide was selected as the reference peak for the characteristic fingerprint of iridoids and their glycosides in GF, reflecting the overall quality of the iridoid and glycoside fraction.

#### 3.1.2. Establishment of the HPLC Characteristic Component Fingerprint

Precision, stability, and repeatability were validated following the procedures outlined in Section 2.4. Peak 11, which exhibited good resolution and excellent absorbance, was selected as the reference peak (Table S4). In the precision, stability, and repeatability tests, the RSD values for the relative retention time (RRT) were 0.02–0.41%, 0.16–4.63%, and 0.23–0.87%, respectively. The RSD values for the relative peak area (RPA) were 0.13–4.89%, 0.02–0.39%, and 0.16–4.63%, respectively. These results indicate that the established HPLC fingerprinting method demonstrates good precision, stability, and repeatability.

Similarity evaluation can be used to classify samples based on correlation coefficients, where values closer to 1 indicate better similarity or quality consistency [21]. To investigate the quality consistency of GF, similarity evaluation was performed after multi-point calibration and full-spectrum peak matching. The fingerprint similarity among the 42 batches of samples (excluding sample S11) ranged from 0.924 to 1. Additionally, the similarity between the fingerprint of each batch and the reference fingerprint ranged from 0.962 to 1. These results indicate that the established HPLC fingerprint is reliable and suitable for the comprehensive quality evaluation and control of GF. Furthermore, the chemical composition of iridoids and their glycosides in samples from different geographical origins was largely consistent, with only minor variations (Table S5).

#### 3.1.3. Validation of Characteristic Peaks

Chromatographic data from GF samples collected at different harvesting times (coded TH1–TH7) were analyzed. The number of characteristic peaks, total peak area of the characteristic peaks, individual peak area of each characteristic peak, retention time, and the percentage of peak area contributed by each characteristic peak were recorded to examine patterns in the individual peak area percentages. Information from the seven harvest time points was compiled (Table S6). Across all samples, Peak 11 consistently accounted for a high proportion of the peak area within the iridoid and glycoside fraction, and the characteristic peaks remained within a stable range (Figure 2B). This demonstrates the stability and reliability of the method.

#### 3.1.4. Results of Reference Peak (Geniposide) Content Determination

The moisture content (%) of each test sample was calculated in accordance with the moisture requirements for GF specified in the Chinese Pharmacopoeia (2025 edition) (Table S7). Following the chromatographic conditions described in Section 2.5.1, the geniposide content in dried GF samples from different geographical origins and batches was determined, and the results complied with the specifications of the Chinese Pharmacopoeia (Table S8).

#### 3.1.5. Establishment of the Quality Control Coefficient *Q* Based on the “Functional Component Group”

Building upon the established efficacy chemical component group fingerprint and referencing the calculation methods for content determination, a mathematical model for the Quality Control Coefficient (*Q*) was constructed.

The calculation method is:

(1) $$\frac{{A\;}_{\mathrm{sum}}}{{M\;}_{\mathrm{injection}}}\;=\;Q\frac{{A\;}_{\min}}{{m\;}_{\mathrm{injection}}}\;$$

After sorting out:

(2) $$Q\;=\;0.01647\;\times \;\frac{A_{\mathrm{sum}}}{{(1-\mathrm{moisture})\;A}_{\min}}$$

Multiplying the small value in Formula (2) by 100 gives:

(3) $$Q\;=1.647\;\times \frac{A_{\;\mathrm{sum}}}{\;{(1-\mathrm{moisture})\;A}_{\min}}$$

*M* is the sampling amount of the test sample; *M*_injection_ is the injection volume of the test sample; *A*_sum_ is the total peak area of the characteristic peaks corresponding to the injection of *M*; *m* is the minimum content of the reference standard in the test sample (*M*), calculated according to the pharmacopoeia’s qualified standard for the index reference peak; *m*_injection_ is the injection volume of *m*; *A*_min_ is the total peak area of the characteristic peaks corresponding to the injection of *m*; (1 − moisture) indicates that the moisture content needs to be subtracted during calculation.

We obtained the minimum content using the following method:

Following the sample preparation method described in Section 2.3.2 and under the conditions specified in Section 2.5.1, a test sample solution prepared from 0.3 g of the medicinal material was injected (10 µL) for HPLC analysis. The minimum content of the index component in the dried GF product specified in the Chinese Pharmacopoeia (2025 edition) (here referring to the minimum geniposide content of 1.8%) was used as the standard to determine the minimum content of the reference index in the sample. This indicates that a sample is considered qualified when the minimum geniposide content in 1 g of the dried sample is 0.01647 g (i.e., 16.47 mg).

#### 3.1.6. Selection of the Quality Control Coefficient *Q* Based on the “Efficacy-Functional Chemical Component Group”

The quartile method was employed for the statistical analysis of *Q* values from 43 batches of GF samples. The lower quartile was selected as the minimum standard for evaluating the quality of the iridoid and glycoside fraction. This is defined such that when analyzing the active iridoid glycoside fraction of a test sample, if its *Q* coefficient reaches or exceeds this threshold, the sample is considered qualified. Furthermore, a higher *Q* value indicates a higher content of the iridoid and glycoside fraction, demonstrating that the *Q* coefficient can simultaneously reflect both the authenticity and the quality grade of the sample.

The *Q* values for the 43 batches of GF samples were calculated by substituting the data into Formula (3), and the results are presented in Table 2. The average *Q* value for samples from Fujian was 4.08, that for Hunan was 4.24, that for Jiangxi was 3.91, and that for Henan was 3.76, as shown in Figure 2C. The *Q* values from different origins were analyzed using the screening method outlined in Section 3.1, and the results are presented in Figure 2D. The lower quartile (*Q* = 3.67) was selected as the minimum quality control standard for the iridoid and glycoside fraction of GF. A higher *Q* value signifies a greater concentration of iridoids and their glycosides. Therefore, in the evaluation of the iridoid and glycoside fraction, if *Q* ≥ 3.67, it indicates that the overall quality of the GF sample is superior.

Based on the content determination results from “3.1.3” and in accordance with the content standard of the Chinese Pharmacopoeia (which requires a geniposide content no less than 1.8%), the 43 batches of samples were classified: Qualified samples were defined as “1”, and unqualified samples as “0”. Similarly, using *Q* = 3.67 as the threshold, the samples were again classified as qualified (“1”) or unqualified (“0”). A visual analysis of this data was performed, and the results are presented in Figure 2E. It presents a comparison between qualified samples screened using the *Q*-value method—which reflects the overall content based on the reference peak—and those selected according to the Chinese Pharmacopoeia content detection standard (geniposide content ≥ 1.8%). Using the Quality Control Coefficient *Q* for screening, 31 batches were deemed qualified based on the overall quality of iridoid and glycoside components, as indicated by geniposide levels. In contrast, according to the Chinese Pharmacopoeia standard, all 43 batches of GF samples qualified based solely on geniposide content. Subsequently, given that *Q* was derived from the total area of the selected peaks and the minimum value based on geniposide, we next explored the relationship between the content of geniposide itself and *Q*. The results of the correlation analysis showed a significant positive correlation between the *Q* value and the geniposide content (*p* < 0.05) (Figure S1A).

MTT assay results showed that at the same administration concentration, the optical density (OD) values followed the order: CON > HQG > QG > M. Significant differences were observed between HQG and M (*p* < 0.001) and between HQG and QG (*p* < 0.05), while no significant difference was found between QG and M (Figure 2F). The results indicated that HQG significantly increased cell viability and was superior to the QG group.

### 3.2. Quantitative HPLC Analysis of Major Functional Components

#### 3.2.1. Methodological Validation Results

Linear equations were obtained through regression analysis using peak area (PA) as the ordinate (*Y*) and concentration (mg/mL) as the abscissa (*X*). The correlation coefficients for the nine compounds ranged from 0.9992 to 1.0000. The results indicated that crocin-I, crocin-II, crocin-IV, crocetin, crocin-III, geniposidic acid, geniposide, genipin-1-O-gentiobioside, and genipin exhibited good linear relationships within the concentration ranges of 1.72–386.75, 2.84–69.37, 6.55–84.22, 5.43–79.16, 0.35–84.22, 53.27–143.07, 55.61–371.47, 103.30–311.00, and 74.90–1541.44 µg/mL, respectively. The precision test results showed relative standard deviation (RSD) values of 0.23–1.06% for the nine components, indicating good instrument precision. In the repeatability assessment, the RSD values for the PA of the nine components were 0.67–2.01%, demonstrating good instrument repeatability. Stability tests revealed RSD values of 0.40–3.36% for the PA of the nine components, confirming that the test solutions remained stable at room temperature for up to 24 h. Furthermore, in the spike recovery experiment, the RSD values for the PA of the nine components were 0.15–1.14%, with recoveries ranging from 98.73% to 103.23%. These results meet established standards, indicating good accuracy of the analytical method (Table S9).

#### 3.2.2. Analysis of Functional Component Differences in GF from Different Origins

**Figure 3:**
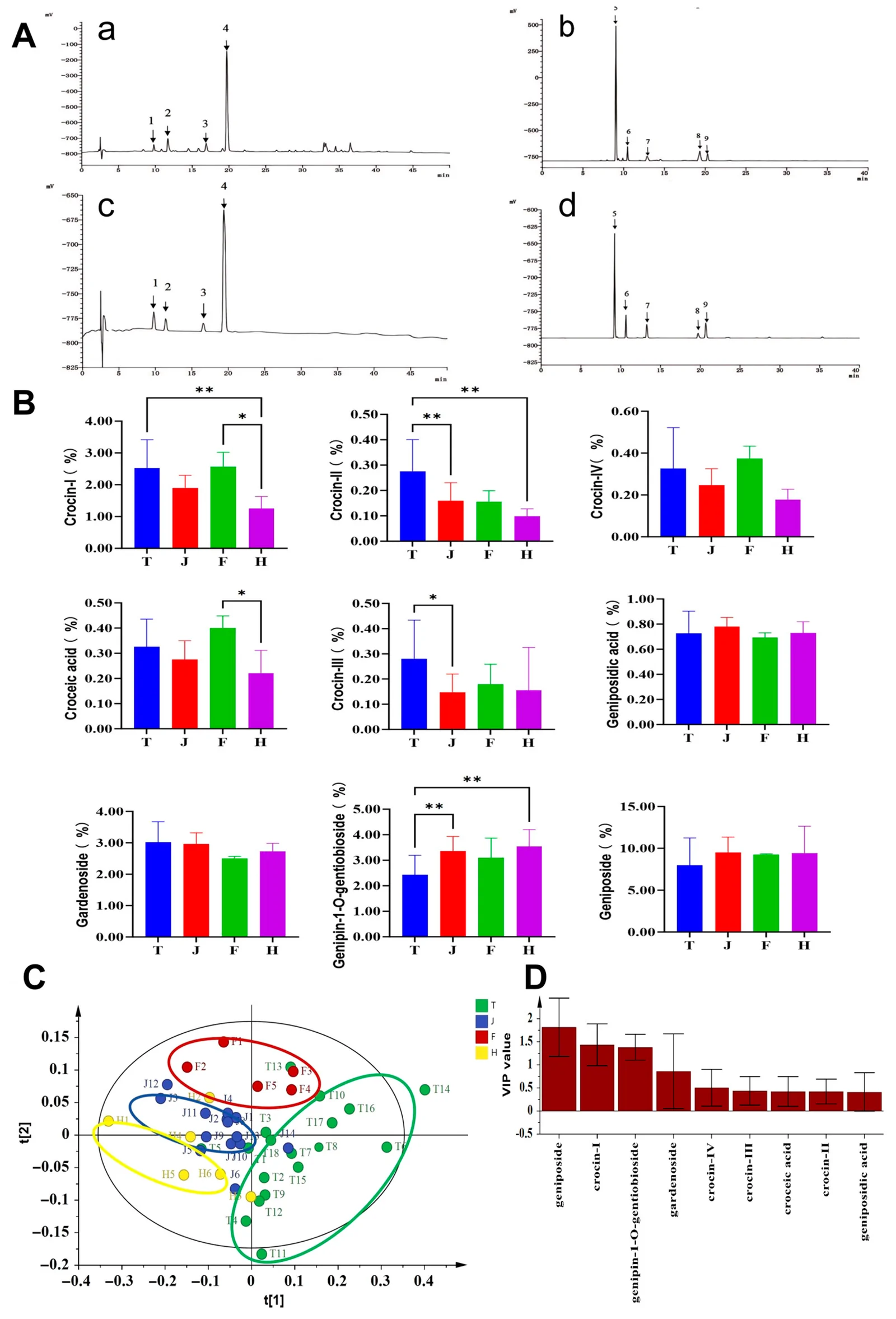
(**A**) Chromatograms of GF samples (**a**,**b**) and mixed standard solution (**c**,**d**) (1: geniposidic acid; 2: gardenoside; 3: genipin-1-O-gentiobioside; 4: geniposide; 5: crocin-I; 6: crocin-II; 7: crocin-IV; 8: croceic acid; 9: crocin-III). (**B**) Intergroup differences in chemical composition among different geographical origins. (**C**) OPLS-DA analysis results of GF from different origins. (**D**) VIP value plot. * *p* < 0.05, ** *p* < 0.01. T, J, F, and H represent Henan, Jiangxi, Fujian, and Henan in China, respectively.

The chromatogram is shown in the figure, and the sample results are presented in the table (Figure 3A, Table S10). In the GF medicinal material samples, the geniposide content ranged from 1.38% to 14.15%. The geniposidic acid content ranged from 0.24% to 0.89%, gardenoside from 1.13% to 3.79%, genipin-1-O-gentiobioside from 0.57% to 4.60%, crocin-I from 0.67% to 3.88%, crocin-II from 0.05% to 0.56%, crocin-IV from 0.11% to 0.89%, croceic acid from 0.05% to 0.54%, and crocin-III from 0.07% to 0.63%.

To provide a more intuitive comparison of the differences in medicinal material content among various origins, mean values were used for comparison. Samples from Jiangxi Province exhibited relatively higher geniposide content (9.50%), as well as a higher average geniposidic acid content (0.78%). Samples from Henan Province showed elevated gardenoside levels (3.02%). Additionally, Henan samples contained higher crocin-II content (0.28%), and samples from Fujian Province exhibited increased crocin-IV content (0.37%). Fujian Province samples demonstrated higher crocetin content (0.40%), whereas Henan samples had elevated crocin-III content (0.29%). These findings indicate that the chemical composition of GF varies across different production regions. To analyze quality differences in GF from various origins, intergroup difference analysis of chemical component indicators was conducted using SPSS 23.0 software. The results revealed significant differences in chemical composition among the origins. Specifically, crocin-I content showed a highly significant difference (*p* < 0.01) between samples from Henan and Hunan, and a significant difference (*p* < 0.05) between samples from Fujian and Hunan. Samples from Henan Province contained relatively higher levels of crocin-II and crocin-III, at 0.276 ± 0.125% and 0.286 ± 0.153)%, respectively. The crocin-II content in Henan samples exhibited a highly significant difference (*p* < 0.01) compared to samples from Jiangxi and Hunan, while crocin-III content showed a significant difference (*p* < 0.01) compared to samples from Jiangxi. Samples from Hunan Province had relatively high genipin-1-O-gentiobioside content, at 3.541 ± 0.661%, with a highly significant difference (*p* < 0.01) observed between Henan samples and those from Jiangxi and Hunan. These statistical analyses demonstrate significant variations in the chemical composition of GF from different geographical origins (Figure 3B and Table S11).

The quantitative results of the nine chemical components in GF from different geographical origins were imported into SIMCA 14.1 software for OPLS-DA analysis (Figure 3C). The model was then subjected to 200 permutation tests to obtain a permutation validation plot. The resulting values were *R*^2^ = 0.0381 and *Q*^2^ = −0.25. The negative *Q*^2^ value indicates that the model does not exhibit overfitting (Figure S1B). The cross-validation results showed that in the PLS-DA model, the cumulative contribution rates were *R*^2^*X* = 0.873 and R^2^Y = 0.297, and the cross-validated *Q*^2^ (cum) value was 0.194, indicating that the model is usable (Figure S1C,D). The overall distribution of the 43 batches of medicinal materials shows that samples from Henan Province cluster into one group, those from Hunan Province cluster into another, samples from Fujian Province form a separate cluster, and those from Jiangxi Province constitute a distinct cluster. Using a VIP value > 1 as the criterion, the differential components preliminarily identified among different origins were geniposide, genipin-1-O-gentiobioside, and crocin-I (Figure 3D). To provide a more comprehensive evaluation of GF samples from different origins, SPSS 23.0 software was used. The content data of the nine chemical components were standardized and used as variables to perform a comprehensive ranking of the 43 batches of samples. Using the criterion of eigenvalue > 1, the model automatically selected two principal components, with a cumulative variance contribution rate of 72.81%. This high representativeness indicates that these two principal components effectively captured most of the information from the original variables. The eigenvalues and variance contribution rates of the correlation matrix, the principal component loading matrix, and the principal component vector values are detailed in Table S12. Based on the coefficients of the eigenvectors, the expressions for the two principal component comprehensive evaluation equations can be established as follows:

Z1 = 0.892X1 + 0.889X2 + 0.853X3 + 0.832X4 + 0.831X5 − 0.086X6 + 0.088X7 − 0.445X8 − 0.002X9

Z2 = −0.077X1 − 0.050X2 − 0.125X3 + 0.185X4 − 0.217X5 + 0.901X6 − 0.855X7 + 0.692X8 + 0.719X9

X1–X9 are crocin-I, crocin-II, crocin-IV, croceic acid, crocin-III, geniposidic acid, gardenoside, genipin-1-O-gentiobioside, and geniposide, respectively.

Z = 0.598Z1 + 0.402Z2

The comprehensive scores (Z) and ranking results are presented in Table S13. Among all samples, the GF samples from Henan origin, namely T16, T17, and T13, ranked in the top three positions.

### 3.3. Analysis of Volatile Components in GF

#### 3.3.1. Analysis of Differences in Volatile Components Among GF from Different Origins

**Figure 4:**
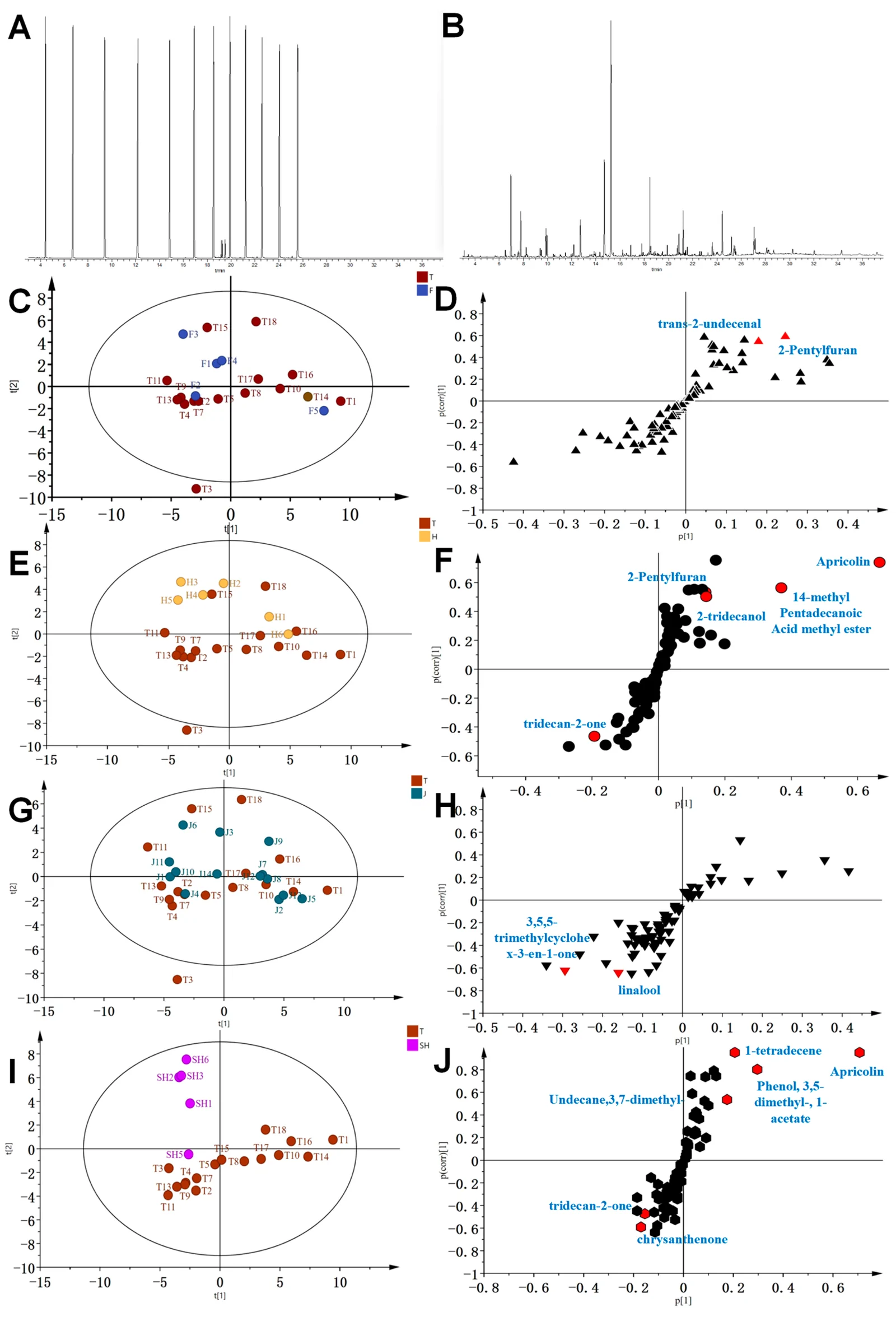
(**A**) Total ion chromatogram of the C8–C20 n-alkane standard solution. (**B**) Total ion chromatogram of GF samples. (**C**) PCA results for Henan-origin vs. Fujian-origin samples. (**D**) S-plot for Henan-origin vs. Fujian-origin samples (components highlighted in red indicate characteristic differential compounds identified through screening with VIP > 1 and *p* < 0.05). (**E**) PCA results for Henan-origin vs. Hunan-origin GF samples. (**F**) S-plot for Henan-origin vs. Hunan-origin GF samples (components highlighted in red indicate characteristic differential compounds identified through screening with VIP > 1 and *p* < 0.05). (**G**) PCA results for Henan-origin vs. Jiangxi-origin GF samples. (**H**) S-plot for Henan-origin vs. Jiangxi-origin GF samples (components highlighted in red indicate characteristic differential compounds identified through screening with VIP > 1 and *p* < 0.05). (**I**) PCA results for Henan-origin GF vs. GVR samples. (**J**) S-plot for Henan-origin GF vs. GVR samples (components highlighted in red indicate characteristic differential compounds identified through screening with VIP > 1 and *p* < 0.05). In the scores plot, the horizontal axis, t[1], represents the scores for the first principal component, indicating the magnitude of its contribution to sample variation. The vertical axis t[2] represents the scores for the second principal component.The red triangles, circles, and hexagons in the figure represent the differential volatile components obtained from preliminary exploration for model construction.

In this study, AMDIS software was used to process the Total Ion Chromatogram (TIC), remove interference peaks, and obtain mass spectral information. Accurate identification of compounds was achieved by combining this with the Retention Index (RI) method [22]. Relative quantification was performed using the peak area normalization method, enabling the comparison of volatile components among GF from different origins (Table S14). The total ion chromatogram of the C8–C20 n-alkane standard solution and the total ion chromatogram of the volatile components in the GF sample are shown in the figure (Figure 4A,B). A total of 78 components were identified, primarily covering aldehydes, esters, and alcohols, among others. The results showed that (E,E)-2,4-decadienal had a relatively high content in GF from all provinces (Henan, 9.58%; Jiangxi, 13.44%; Fujian, 9.31%; Hunan, 9.81%; GVR, 5.24%), indicating it is a key volatile component of GF. Additionally, components such as methyl 14-methylpentadecanoate, octadeca-9,12,15-triene, (2E,4E)-2,4-decadienal, linolenyl alcohol, and caryophyllene were also prominent in most samples. There were 23 components in Henan-origin GF with higher contents than in other origins, including 2-tridecanone, methyl linoleate, and 4-fluorobenzyl chloride. In GVR, gamma-nonalactone showed the highest content (12.32%), significantly distinguishing it from samples of other origins. The comparison revealed that in GF, the contents of most components, such as (E,E)-2,4-decadienal, (2E,4E)-2,4-decadienal, methyl 14-methylpentadecanoate, and caryophyllene, were generally higher than in GVR, reflecting differences in their volatile composition. The volatile component profiles of GF from different origins were similar, but the relative contents exhibited regional variations. (E,E)-2,4-Decadienal can serve as a characteristic component of GF, while the high content of gamma-nonalactone suggests that GVR possesses a different volatile material basis.

#### 3.3.2. PCA and S-Plot

The peak area data of 78 volatile compounds detected in samples from Henan, Hunan, and Fujian origins of GF and GVR were analyzed using Principal Component Analysis (PCA) with SIMCA 14.1 software. Patterns and differences among samples were evaluated based on their distribution within this principal component space. The separation between Henan-origin and Jiangxi-origin GF samples was minimal, indicating little variation in volatile components between these two origins (Figure 4G). In contrast, Henan-origin samples were clearly distinguished from those of Hunan-origin, Fujian-origin, and GVR. Henan-origin GF clustered in the lower region of the plot, while Hunan-origin, Fujian-origin, and GVR samples aggregated in the upper region, demonstrating a distinct clustering trend. This suggests significant differences in volatile components between Henan-origin samples and those from the other origins and GVR (Figure 4C,E,I).

To further analyze the differences among different origins, S-plot analysis was conducted based on the PCA results to investigate the characteristic differential components between Henan-origin GF and Hunan-origin GF, and Fujian-origin GF, as well as GVR. Compounds with VIP > 1 and *p* < 0.05 were selected, and S-plot analysis was further combined for preliminary exploration and screening of differential compounds. The characteristic differential components between Henan-origin and Fujian-origin volatile oils were trans-2-undecenal and 2-Pentylfuran (Figure 4D). The characteristic differential components between Henan-origin and Hunan-origin volatile oils were 2-Pentylfuran, Apricolin, 14-methyl Pentadecanoic Acid methyl ester, 2-tridecanol, and tridecan-2-one (Figure 4F). The characteristic differential components between Henan-origin and Jiangxi-origin volatile oils were 3,5,5-trimethylcyclohex-3-en-1-one and linalool (Figure 4H). The characteristic differential components between Henan-origin GF and GVR volatile oils were 1-tetradecene, Phenol, 3,5-dimethyl-, 1-acetate, Apricolin, Undecane, 3,7-dimethyl-, Tridecan-2-one, and Chrysanthenone (Figure 4J). The compound that showed significant differences in volatile oils between Henan-origin GF and both the Jiangxi and Fujian reference origin medicinal materials was 2-Pentylfuran. The compound that showed significant differences in volatile oils between Henan-origin GF and both the Hunan reference origin medicinal material and GVR was tridecan-2-one. The results of the 200 permutation tests indicated that the established model was reliable (Figure S1E–H).

### 3.4. Analysis of Inorganic Element Content in GF

#### 3.4.1. Characteristics of Inorganic Element Content in GF Peel and Kernel

**Figure 5:**
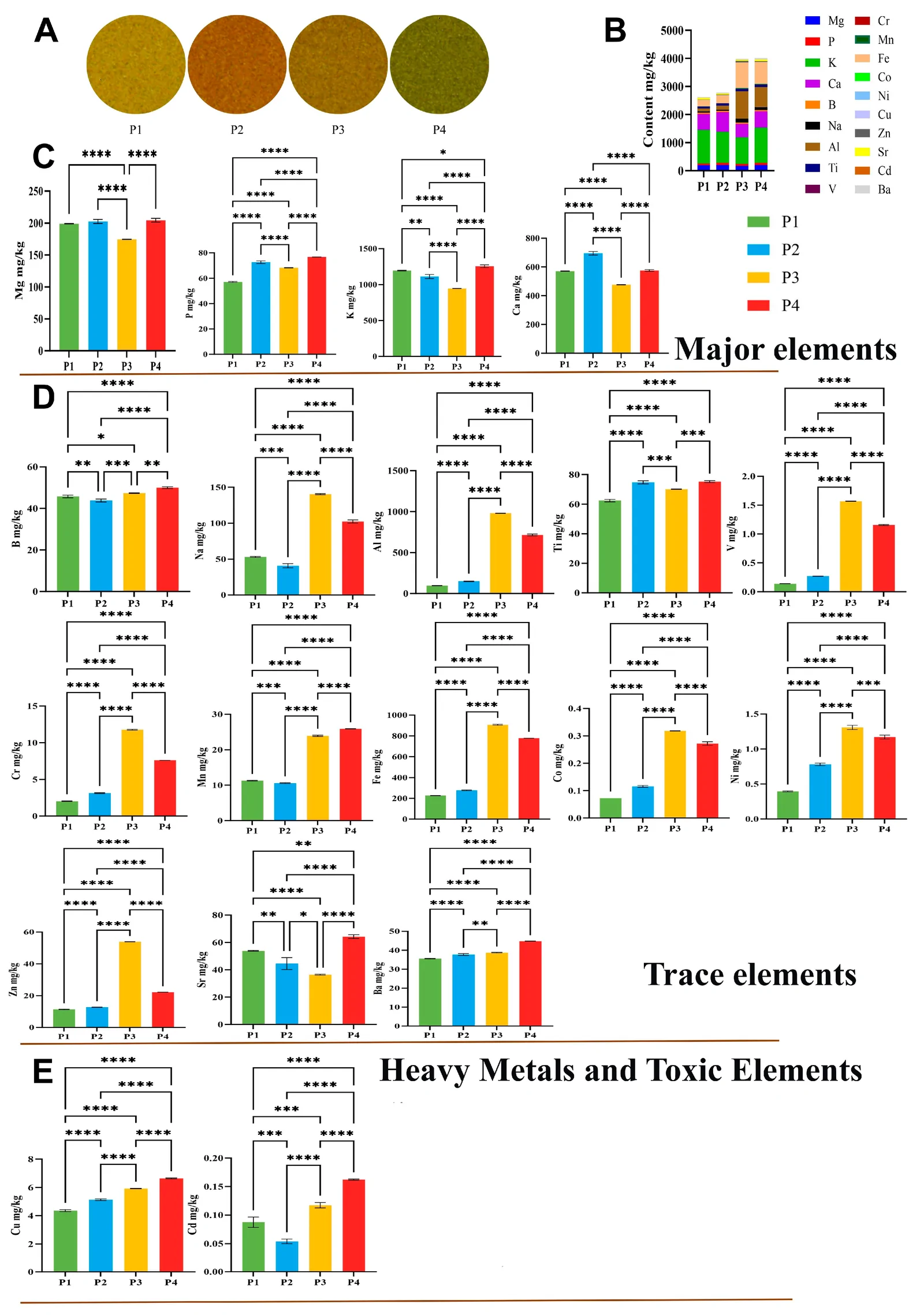
(**A**) Classification diagram of GF peel samples at the mature stage. (**B**) Differences in inorganic element content among different batches of GF peel. (**C**) Intergroup differences in macronutrient element content among different peel groups. (**D**) Intergroup differences in trace element content among different peel groups. (**E**) Intergroup differences in heavy metal and harmful element content among different peel groups. * *p* < 0.05, ** *p* < 0.01, *** *p* < 0.001, and **** *p* < 0.0001.

**Figure 6:**
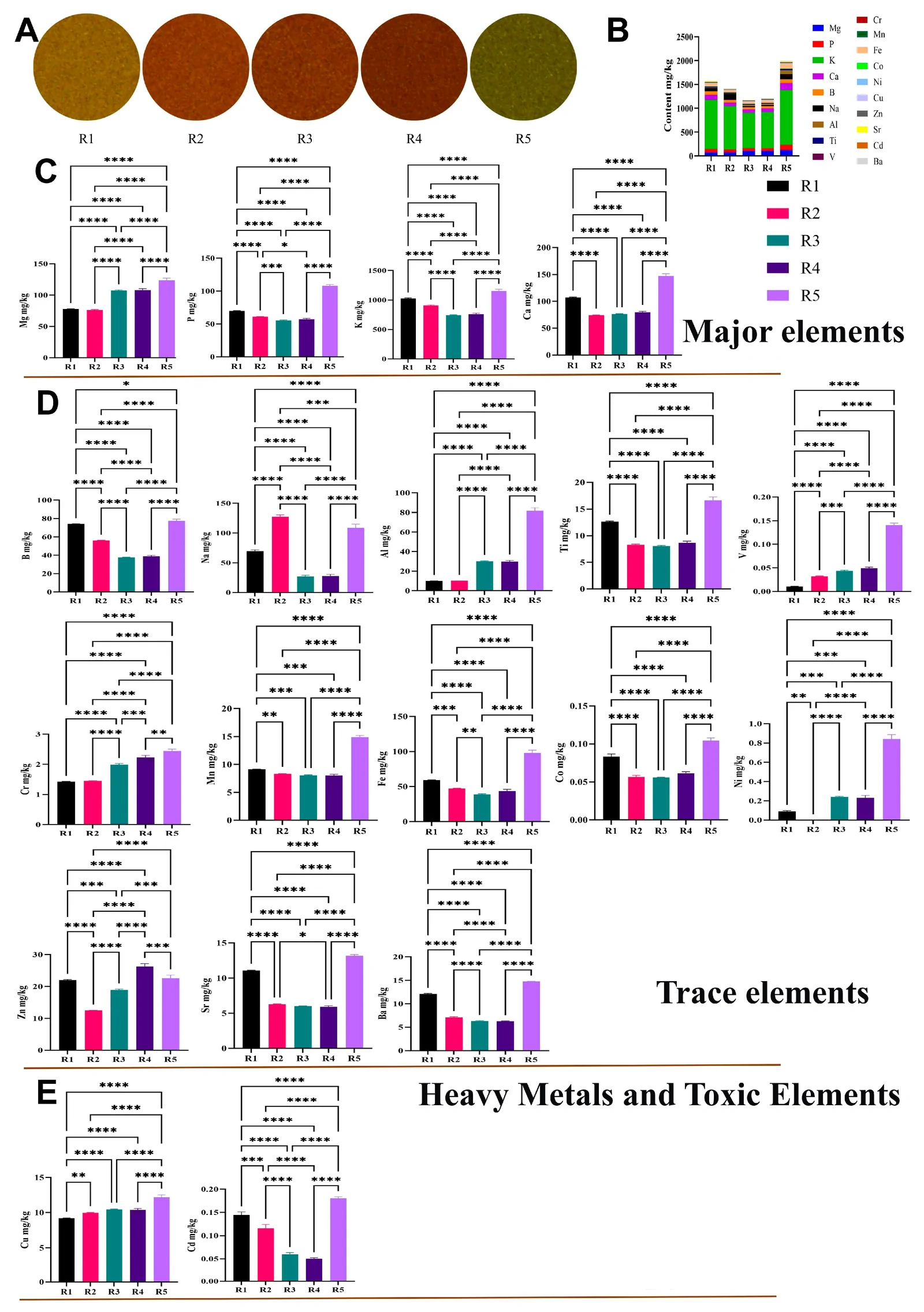
(**A**) Classification diagram of GF kernel samples at the mature stage. (**B**) Differences in inorganic element content among different batches of GF kernels. (**C**) Intergroup differences in macronutrient element content among different kernel groups. (**D**) Intergroup differences in trace element content among different kernel groups. (**E**) Intergroup differences in heavy metal and harmful element content among different kernel groups. * *p* < 0.05, ** *p* < 0.01, *** *p* < 0.001, and **** *p* < 0.0001.

The peel and kernel of GF fruits harvested at maturity were separated and classified according to color, from light to dark. The classification groups for peel and for kernel samples are shown in Figure 5A and Figure 6A. Each batch was analyzed in triplicate. The contents of 22 inorganic elements were determined using ICP-MS. As, Hg, and Pb were not detected in any of the measured samples. The data were visualized and analyzed (Table S15). The linearity range was between 0.9996 and 1.0000. The RSD values for precision, stability, repeatability, and accuracy experiments were all less than 3%, indicating good performance of the instrument, test samples, and method (Table S16). The total content of 19 inorganic elements in GF peel followed the order: P4 > P3 > P2 > P1 (Figure 5B). The macronutrient K showed the highest content in sample P4, averaging 1255.54 mg/kg, significantly higher than in other samples. Ca was relatively high in sample P2, averaging 695.19 mg/kg, suggesting differences in calcium storage among peels of different colors or from different parts (Figure 5C). The distribution of trace elements exhibited significant sample specificity. Fe content was extremely high in P3, averaging 906.73 mg/kg, far exceeding that in other samples. Al also showed abnormal enrichment in P3, averaging 980.60 mg/kg. Zn content was highest in P3, averaging 53.89 mg/kg, which was more than double that in other samples. Additionally, the trace elements Na, Cr, V, Ni, and Co were present in higher amounts in sample P3 (Figure 5D). The heavy and harmful elements Cu and Cd were higher in sample P4, with noticeable differences among samples (Figure 5E).

The total content of inorganic elements in GF kernels followed the order: R5 > R1 > R2 > R4 > R3 (Figure 6B). Among the macroelements, K showed the highest content, reaching an average of 1150.85 mg/kg in sample R5, significantly higher than in other samples. P was highest in R5, averaging 108.08 mg/kg, approximately 1.5–2 times that of other samples. Mg and Ca were also at relatively high levels in R5, at 123.83 mg/kg and 147.29 mg/kg, respectively. This sample represents brown kernels, which do not comply with the appearance characteristics specified in the Chinese Pharmacopoeia (2025 edition) (Figure 6C). Among the trace elements, Fe content was highest in R5, averaging 98.11 mg/kg, followed by R1 at 59.11 mg/kg. Zn content was relatively high in sample R4, averaging 26.26 mg/kg, showing a significant difference from other samples (Figure 6D). Among the heavy metal elements, Cu content was relatively stable across all samples, with a slightly higher level in R5, averaging 12.18 mg/kg. Cd content was generally low, slightly higher in R5 at an average of 0.18 mg/kg, while other samples were all below 0.15 mg/kg (Figure 6E).

#### 3.4.2. OPLS-DA

**Figure 7:**
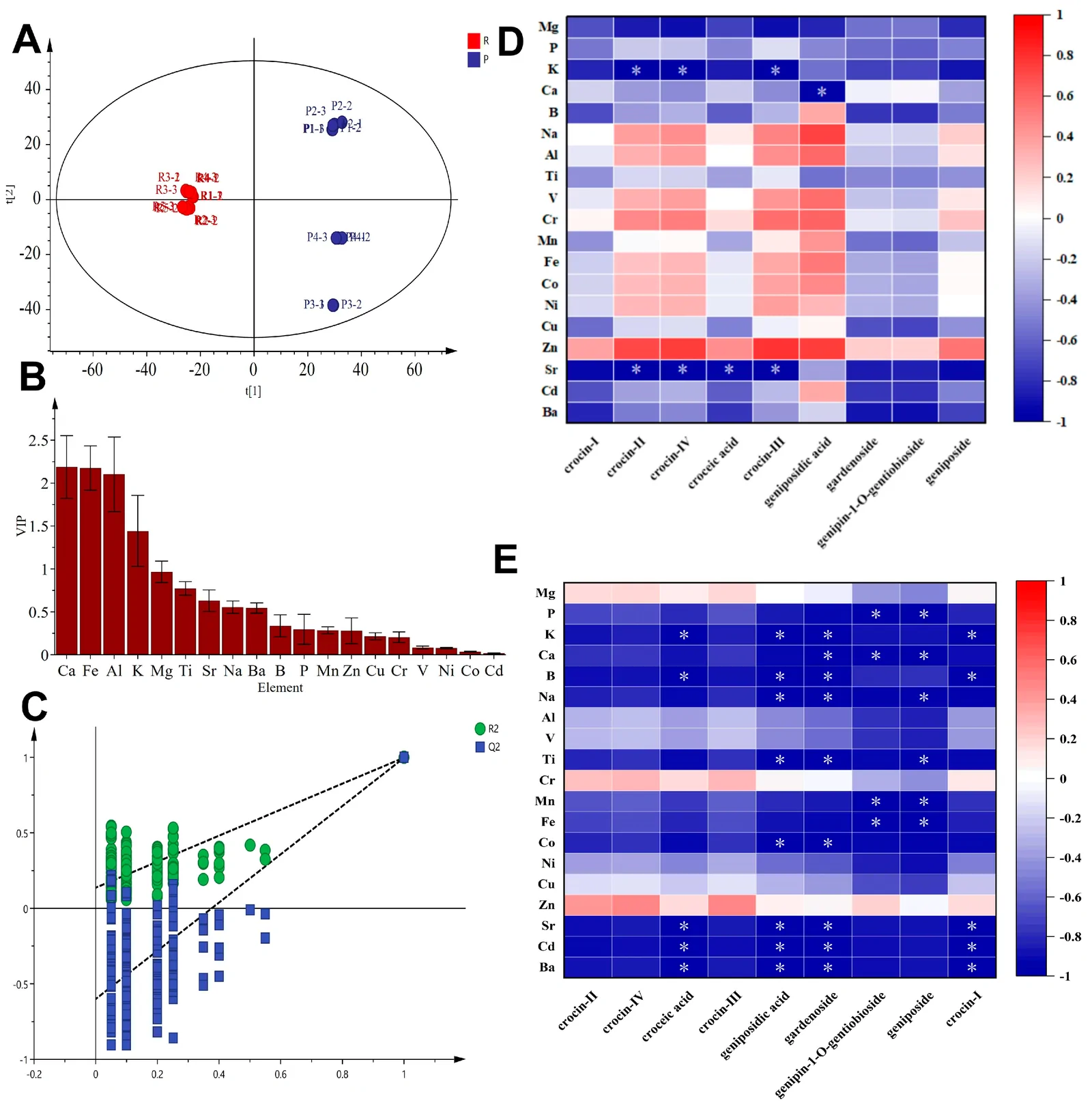
(**A**) OPLS-DA results for peel and kernel. The horizontal coordinate t[1] represents the magnitude of the regression coefficient weights for the predictive principal component, and the vertical coordinate t[2] represents the magnitude of the regression coefficient weights for the orthogonal principal component. (**B**) VIP value results. (**C**) Results of the 200-permutation test. (**D**) Correlation analysis results between chemical components and inorganic elements in the peel. (**E**) Correlation analysis results between chemical components and inorganic elements in the kernel. The asterisk (*) indicates a significant correlation (*p* < 0.05).

To further investigate the characteristic differential elements between GF peel and kernel, SIMCA 14.1 software was used to perform OPLS-DA analysis on the content of 19 inorganic elements (excluding As, Hg, and Pb) in 12 batches of peel and 15 batches of kernel samples. The established model had values of *R*^2^*X* = 0.988, *R*^2^*Y* = 0.994, and *Q*^2^ = 0.992, all greater than 0.5. A permutation test with 200 iterations was conducted to validate its accuracy. The closer *R*^2^*Y* is to 1 and *Q*^2^ is to 1, the more stable and reliable the model is (Figure 7C). The peel and kernel samples of GF were clearly separated into two categories, indicating differences in the content of inorganic elements rich in the peel and kernel samples from Tanghe County, Henan Province (Figure 7A). Ca, Fe, Al, and K were preliminarily screened as the quality-differentiating characteristic elements among the inorganic elements in GF peel and kernel (Figure 7B).

#### 3.4.3. Correlation Analysis

Based on preliminary research data (Table S17), an exploratory correlation analysis was performed using ORIGIN software between nine characteristic components and 19 inorganic elements (excluding As, Hg, and Pb) in the peels and kernels of GF. Overall, the correlation coefficients between chemical components and inorganic elements in the peel ranged from −0.998 to 0.781, while in the kernel they ranged from −0.999 to 0.485, with both showing a significant negative correlation trend. In the peel, most crocin components exhibited significant negative correlations with inorganic elements. Crocin-II and crocin-IV showed significant negative correlations with K and Sr (*p* ≤ 0.05). Crocetin and crocin-III also showed significant negative correlations with Sr (*p* ≤ 0.05). Notably, crocin-III exhibited a highly significant negative correlation with K (*p* ≤ 0.01) (Figure 7D). Among the iridoids, geniposidic acid also showed a significant negative correlation with Ca (*p* ≤ 0.05). In the kernel, crocin-I and crocetin showed significant negative correlations with elements such as K, B, Sr, Ba, and Cd (*p* ≤ 0.05). Crocin-I exhibited a highly significant negative correlation with Cd (*p* ≤ 0.01). Iridoids overall also showed negative correlations. Specifically, geniposidic acid showed significant negative correlations with B, Na, Ti, and Co, and highly significant negative correlations with K, Sr, Ba, and Cd. Gardenoside, geniposide, and genipin-1-O-gentiobioside also showed significant or highly significant negative correlations with various elements (Figure 7E).

## 4. Discussion

The application of fingerprinting technology to evaluate the quality of natural medicinal raw materials can characterize the intrinsic components of traditional Chinese medicines [23] and reflect their inherent quality [24]. However, current single-indicator standards and traditional fingerprint similarity analysis can hardly achieve quantitative grading of overall quality. Therefore, functional component groups and quantitative grading models are required.

The screening and validation of the active phytonutrient group of iridoid glycosides constitute the core foundation for establishing the quality evaluation system in this study. GF contains two primary functional component groups: iridoid glycosides and crocins. Among these, the iridoid glycosides are closely associated with the key biological activities of GF, such as antioxidant and anti-inflammatory effects. From the perspective of classical herbal texts, in the clinical application of GF within traditional Chinese medicine, the iridoids and their glycosides are associated with the core therapeutic effects of “clearing heat, draining fire, and relieving restlessness.” Therefore, they serve as the core indicators for quality evaluation. In contrast, the therapeutic effects corresponding to the crocins are not documented in traditional herbal literature. They represent the crucial material basis supporting the various therapeutic functions of GF beyond its coloring properties [25]. In this study, LC-MS identified nine iridoid glycoside-related components in GF, accounting for 85% of the total peak area of the extracted fraction. Fifteen absorption peaks were screened using geometric mean, positional mean, and quartile methods. Seven stable characteristic peaks were selected, accounting for 80.02% of the total iridoid glycoside peak area; among them, four core peaks (Peaks 2, 6, 9, and 11) were stable and represented the most stable, highest-area subset. Multi-method screening reduced the bias of single statistical methods. Method validation showed acceptable RSDs for relative retention times and relative peak areas, and fingerprint similarities of 42 GF batches ranged from 0.924 to 1, indicating good precision, stability, and repeatability. This method enables qualitative characterization of the overall chemical composition of the core functional components in GF, surpassing single-indicator methods.

The innovative proposal and validation of the quality control coefficient *Q* model mark a significant breakthrough in quantitative quality evaluation of GF. The *Q* qualification criterion (*Q* ≥ 3.67) does not conflict with the Chinese Pharmacopoeia geniposide standard (≥1.8%). *Q* was developed by incorporating this pharmacopoeial limit. Using 0.3 g of raw material for extraction and an injection volume of 10 μL, the minimum geniposide content in a qualified sample was calculated as 0.01647 g/g (16.47 mg/g). Thus, *Q* ≥ 3.67 is a grading threshold based on the pharmacopoeial standard and further incorporates the total peak area information of seven characteristic peaks in the iridoid glycoside fraction. The pharmacopoeia provides a single-indicator qualification criterion: geniposide ≥1.8% means qualified. In contrast, *Q* uses the total peak area of the seven characteristic peaks and a mathematical model to grade samples. Higher *Q* indicates greater overall abundance of the functional component group. *Q* therefore elevates quality control beyond pharmacopoeial standards and provides an operational chemical grading standard for raw materials. Among the seven peaks, four core peaks account for 68.18%, and the remaining three account for 11.83%. Because low-response peaks contribute little to total peak area, measurement drift minimally interferes with *Q*. Preliminary experiments with different integration parameters and experimenters showed repeatable grading and a stable model. Future studies should expand sample size and validate across multiple batches. Using this threshold, 31 batches were identified as high-quality, while all 43 batches met the pharmacopoeial geniposide standard. These results show that *Q* can distinguish “qualified” from “high-quality” GF raw materials while complying with pharmacopoeial standards, partly addressing the industry challenge that single indicators fail to reflect overall quality differences. The *Q* model centers on the active phytonutrient group and includes moisture content, enhancing scientific rigor and practical value. It further illustrates regional GF quality variation and provides a quantifiable reference for the food industry when selecting high-quality GF raw materials.

Compared with existing studies on the quality evaluation of GF, the research by Dong primarily focused on the screening of quality markers for GF and established a method for the rapid discovery of these markers; however, it did not succeed in constructing a quantitative grading system for GF quality [26]. While Zhu achieved geographical origin traceability of GF using metabolomics techniques, the study did not integrate the analysis of volatile components with that of inorganic elements [11]. Existing studies often focus on only a single type of substance or a single evaluation indicator, making it difficult to holistically reflect the overall quality of GF [10]. This study utilizes HPLC to establish a *Q* grading model for iridoid glycosides and their related compounds (associated with traditional Chinese medicine efficacy). The following section presents exploratory and supplementary analyses. GC-MS was used to screen differential volatile markers for geographical origin traceability, with subsequent validation by HS-SPME-GC-MS; ICP-MS was used to analyze characteristic inorganic elements in different plant parts, thereby providing a reference for processing, utilization, and safety evaluation. This part constitutes an exploratory analysis. Differences in the accumulation of elements and components between the pericarp and kernel may arise from tissue specificity and may also be confounded by soil conditions and processing techniques; the relationship between these elements and chemical components still requires further verification.

The results of the cell viability assay indicated that, under LPS-stimulated conditions, HQG exhibited relatively superior performance in maintaining cell viability, which may reflect the potential impact of differences in the chemical abundance of the iridoid glycoside component group among samples with different *Q* values on cellular metabolic activity. This study did not measure NO or inflammatory cytokine levels and only demonstrated that high-*Q* samples possess better cytocompatibility. The association between *Q* grading and the functional activities (e.g., anti-inflammatory, antioxidant) of GF extracts remains to be validated through specific functional indicator assays.

In summary, we have developed a scientific and comprehensive quality evaluation system based on functional component groups and quality control coefficients, enabling both qualitative characterization and quantitative grading of the overall quality of GF. This method effectively addresses the practical demands of the food industry for refined quality control of GF raw materials, aligning with the global trend toward natural, safe, and high-quality food ingredients.

## 5. Conclusions

This study addressed the limitations of current quality evaluation methods for GF as a food ingredient, including reliance on a single evaluation index, failure to comprehensively reflect overall quality, and difficulty in effectively grading raw materials due to diverse geographical origins and complex chemical compositions. Based on seven key characteristic peaks of iridoid glycosides and their related compounds in GF, an HPLC fingerprint was established using the core structure of the “functional component group” as the basis. A mathematical model, the quality control coefficient (*Q*), was proposed, and, on the basis of the Chinese Pharmacopoeia standard, 31 batches met the *Q* threshold of *Q* ≥ 3.67 among 43 batches, enabling quantitative and graded evaluation of the overall quality of raw materials and effectively distinguishing “qualified” from “superior” raw materials. In cell experiments, HQG outperformed QG in the MTT assay of LPS-stimulated RAW264.7 cells. In addition, analyses of volatile components and inorganic elements were included to explore differential volatile markers for geographical origin traceability and key characteristic elements for distinguishing medicinal parts. Preliminary exploration revealed negative correlations between iridoid glycosides, crocins, and various elements. The stable and reliable quality evaluation system for GF raw materials established in this study not only provides a systematic technical solution for the industrial application of GF as a natural food colorant and functional food ingredient but also offers a reference model for the quality evaluation and standardization of other natural food ingredients with complex chemical compositions.

## Figures and Tables

**Table 1:** Peak areas of 15 peaks in the iridoid and iridoid glycoside fraction of *Gardenia jasminoides*.

Peak Number	RT (min)	Unimodal Area (A)	Total Peak Area (A Sum)	Peak Area Proportion (A/A Sum, %)
1	5.907	2,435,295.73	59,023,126.37	4.13
2	7.319	3,554,334.88	59,023,126.37	6.02
3	8.091	2,028,305.11	59,023,126.37	3.44
4	9.032	1,495,527.94	59,023,126.37	2.53
5	10.174	2,034,659.1	59,023,126.37	3.45
6	11.043	3,549,773.54	59,023,126.37	6.01
7	12.014	1,610,038.3	59,023,126.37	2.73
8	15.13	2,016,976.85	59,023,126.37	3.42
9	16.028	2,444,599.57	59,023,126.37	4.14
10	18.377	22,796,15.52	59,023,126.37	3.86
11	18.974	30,698,803	59,023,126.37	52.01
12	32.453	2,266,499.12	59,023,126.37	3.84
13	32.981	450,769.68	59,023,126.37	0.76
14	33.247	204,341.08	59,023,126.37	0.35
15	35.873	1,953,586.95	59,023,126.37	3.31

**Table 2:** *Q* values of GF raw materials from different producing regions.

Sample	*Q* Value	Sample	*Q* Value
T1	2.498	H5	3.87
T2	4.7	H6	2.239
T3	4.636	J1	3.811
T4	4.262	J2	4.261
T5	4.465	J3	3.238
T6	1.385	J4	3.653
T7	2.638	J5	3.389
T8	4.212	J6	3.806
T9	4.272	J7	4.196
T10	2.889	J8	3.046
T11	1.596	J9	4.13
T12	4.32	J10	3.951
T13	4.128	J11	3.636
T14	1.395	J12	3.629
T15	4.17	J13	4.042
T16	4.36	J14	5.282
T17	3.68	F1	2.594
T18	4.297	F2	3.571
H1	4.031	F3	4.241
H2	3.971	F4	4.251
H3	3.948	F5	4.125
H4	3.878		

## Data Availability

The original contributions presented in this study are included in the article/Supplementary Materials. Further inquiries can be directed to the corresponding author.

## Supplementary Materials

The following supporting information can be downloaded at: https://www.mdpi.com/article/10.3390/foods15183346/s1, Figure S1: (A) Correlation results between geniposide and Q; (B) cross-validation results corresponding to Figure 3C; (C) principal component analysis; (D–H) cross-validation results supplementing the S plot in Figure 4 and the inorganic elements in Figure 7A; Tables S1–S17: Detailed data supporting the establishment of coefficient Q in the article, as well as the detailed qualitative and quantitative data obtained by HPLC, GC-MS, and ICP-MS.
