# Supplementary material for: Quality Evaluation Research Based on Functional Component Group and Quality Control Coefficient: A Case Study of Gardenia jasminoides
Source: Foods. 2026 Sep 21;15(18):3346. doi: 10.3390/foods15183346 (PMC13606476; doi:10.3390/foods15183346)
Supplement: Supplementary file 1 [file foods-15-03346-s001.zip › Figure S1.pdf]

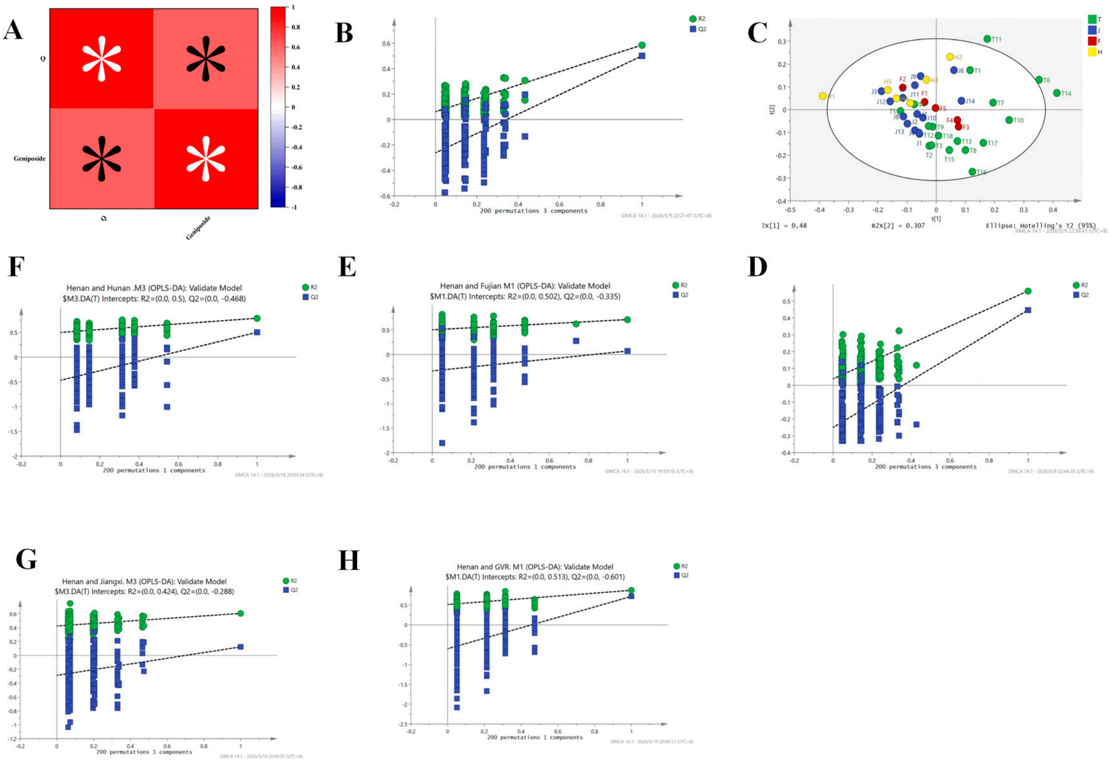

Figure S1. (**A**) Correlation results between geniposide and Q; (**B**) cross-validation results corresponding to Figure 3C; (**C**) principal component analysis; (**D**–**H**) cross-validation results supplementing the S plot in Figure 4 and the inorganic elements in Figure 7A.
